# Evaluation of Spin Columns for Human Plasma Depletion to Facilitate MS-Based Proteomics Analysis of Plasma

**DOI:** 10.1021/acs.jproteome.1c00378

**Published:** 2021-07-28

**Authors:** Xiaofang Cao, AnnSofi Sandberg, José Eduardo Araújo, Filip Cvetkovski, Erik Berglund, Lars E. Eriksson, Maria Pernemalm

**Affiliations:** † Cancer Proteomics Mass Spectrometry, Scilifelab, Department of Oncology and Pathology, Karolinska Institutet, SE-141 86 Stockholm, Sweden; ‡ Research and Development, ITB-Med AB, SE-113 66 Stockholm, Sweden; § Section of Endocrine and Sarcoma Surgery, Department of Molecular Medicine and Surgery; Department of Clinical Science, Intervention and Technology (CLINTEC), Division of Transplantation, Surgery, Karolinska Institute, SE-141 86 Stockholm, Sweden; ∥ Department of Learning, Informatics, Management and Ethics, Karolinska Institutet, SE-171 77 Stockholm, Sweden; ⊥ Medical Unit Infectious Diseases, Karolinska University Hospital, SE-141 86 Huddinge, Sweden; # School of Health Sciences, City University of London, London EC1 V 0HB, United Kingdom

**Keywords:** plasma, high abundant protein depletion, heat-inactivation, biomarkers

## Abstract

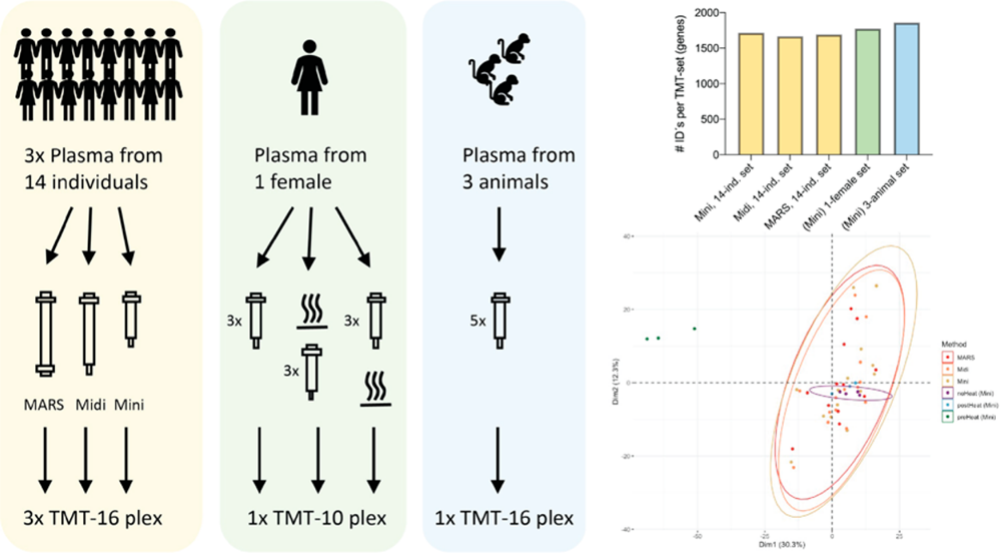

High abundant protein depletion is a common strategy applied to increase analytical depth in global plasma proteomics experiment setups. The standard strategies for depletion of the highest abundant proteins currently rely on multiple-use HPLC columns or multiple-use spin columns. Here we evaluate the performance of *single-use* spin columns for plasma depletion and show that the single-use spin reduces handling time by allowing parallelization and is easily adapted to a nonspecialized lab environment without reducing the high plasma proteome coverage and reproducibility. In addition, we evaluate the effect of viral heat inactivation on the plasma proteome, an additional step in the plasma preparation workflow that allows the sample preparation of SARS-Cov2-infected samples to be performed in a BSL3 laboratory, and report the advantage of performing the heat inactivation postdepletion. We further show the possibility of expanding the use of the depletion column cross-species to macaque plasma samples. In conclusion, we report that single-use spin columns for high abundant protein depletion meet the requirements for reproducibly in in-depth plasma proteomics and can be applied on a common animal model while also reducing the sample handling time.

## Introduction

Analyzing human plasma with modern mass spectrometry (MS)-based proteomics technologies holds enormous potential, not only as a source of biomarkers for disease and treatment, but also to study complex systemic signaling events that are involved in basic biological processes. However, due to a range of major analytical challenges, in-depth plasma proteome studies of large clinical cohorts remain a challenge. One of the main fundamental differences when applying proteomics methods to plasma compared to cellular or tissue material is the presence of a few extremely high abundant proteins that dominate the protein content and hamper the detection of other less abundant protein species. Albumin, for example, has a plasma concentration of 35–50 mg/mL, which can be compared to the clinically used tissue leakage marker Prostate Specific Antigen (PSA) that is present in low ng/mL concentrations. In plasma about 55% of the total protein mass is made up by albumin alone and as few as seven proteins together make up 85% of the total protein mass. This can be compared with estimates from tissue and cellular data where 2300 housekeeping proteins are thought to make up 75% of the protein mass.^1^ In addition, there is an enormous variability between individuals in their composition of the plasma proteome, attributed both to hereditary as well as environmental and temporal factors,^2,3^ making robust measurements and throughput key components in any plasma analysis workflow.

In general, achieving high plasma proteome coverage using MS-based technologies is dependent on extensive fractionation. Consequently, aiming for large proteome coverage comes at the cost of low sample throughput, and vice versa high-throughput comes at the cost of limited proteome coverage. To repetitively identify and quantify a consistent set of proteins across a large number of samples is of particular challenge, and the number of proteins identified across all samples drastically drops in cohorts consisting of more than 100 samples.

With the transition toward personalized medicine, much effort has been put into adapting MS methods to be applicable in the clinical setting, focusing on robustness, throughput, and analytical turnaround time using both data dependent (DDA)^4−6^ and data independent (DIA)^7−9^ analysis methods. In parallel, there is a need for in-depth unbiased global methods for discovery proteomics, enabling discovery of low-abundant, modified and noncanonical variants of proteins in plasma. We have previously developed a method for in-depth plasma proteomics and proteogenomics based high resolution isoelectric focusing (HiRIEF) peptide fractionation in combination with high abundant protein depletion and TMT labeling.^10^ Using the plasma HiRIEF method we show that we can detect low abundant tissue leakage proteins as well as individual specific protein sequence variants transferred across the placenta during pregnancy. During the development of the method, we optimized the MS analysis time to improve the throughput. In addition, we identified the depletion step using multiple-use HPLC columns as bottleneck. High abundant protein depletion using antibodies or other affinity resins is one of the most used prefractionation methods in in-depth plasma proteomics.^11−14^ Removing high abundant proteins in a native setting inevitably coremove additional nontargeted proteins of potential significance. In addition, the step often requires subsequent concentration and buffer-exchange steps and is hence often omitted in studies aiming for high-throughput. However, the benefit on the number of identified proteins is well documented and the depletion step remains widely used in in-depth proteomics.^5,10,15^

Examples of depletion systems include multiple-use HPLC columns for removal of up to 14 proteins,^10,16^ IgY ultra high depletion columns,^15^ and multiple-use spin columns.

HPLC based depletion systems have the benefit of being automated, providing a robust platform for depletion. However, not every laboratory has access to the required equipment and, in addition to the analysis time setting up the system, HPLC washes and maintenance limits the throughput. Multiple-use spin columns for high abundant protein depletion have been available for a long time, with the disadvantage of a reduced throughput due to limitations in parallelization.

Recently, single-use depletion spin columns have been developed (i.e., High Select Top14 Abundant Protein Depletion Mini Spin Columns, Thermo) that greatly reduce the handling time compared to HPLC columns and enable parallel depletion of multiple samples, which has not been possible with the previous multiple-use columns. Also, the COVID-19 pandemic has highlighted the need for a depletion protocol easily adapted in a BSL3 facility without the need for special equipment such as an HPLC system, as heat inactivation of the virus prior to depletion could affect the depletion efficacy by disrupting the targeted epitopes.

**Figure 1:**
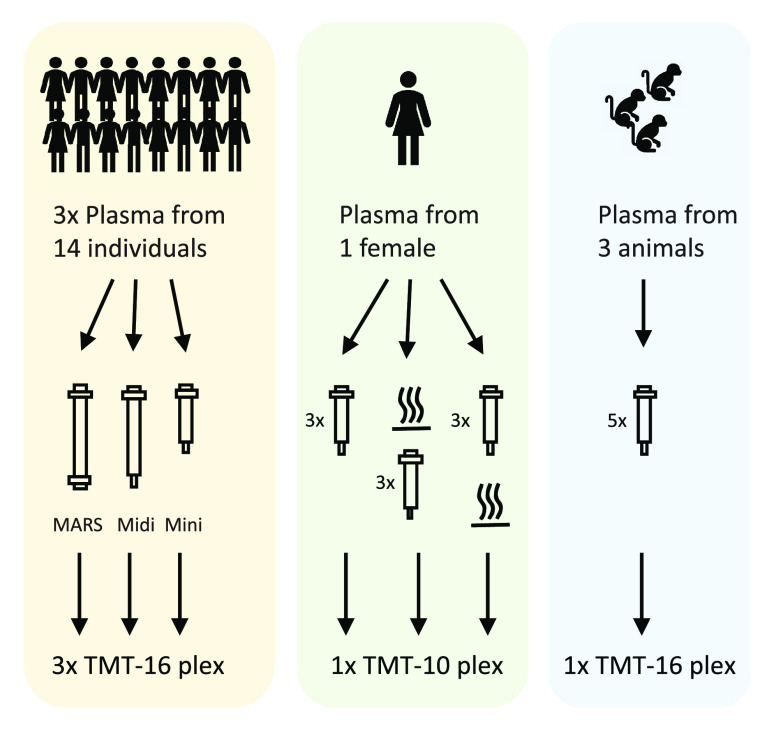
Schematic drawing of workflow(s).

In the present study, we evaluated the use of single-use spin columns for high abundant protein depletion prior to in-depth MS based global plasma analysis, to improve throughput while maintaining high proteome coverage and high reproducibility. We compared the depletion efficacy both in relation to virus heat-inactivation, and cross-species on an animal model (Figure 1).

## Materials and Methods

### Chemicals and Reagents

### Plasma Sampling

#### Human Plasma Samples

This study was carried out according to the Declaration of Helsinki and samples were anonymized to protect the privacy of the study participants. After approval by the Stockholm regional ethics board (EPN: ref no 2014/1290–32), plasma samples were collected from September 2014–November 2015. Peripheral venous blood were collected in EDTA tubes (BD Vacutainer K2E 7.2 mg, BD Diagnostics). EDTA tubes were first centrifuged at 1500*g* at 4 °C for 10 min. The supernatant was transferred to a new tube and centrifuged at 3000*g* at 4 °C for 10 min. The plasma was then aliquoted and kept at −80 °C until analysis. Fourteen plasma samples were randomly chosen for the current plasma depletion methods evaluation.

#### Cynomolgus Macaque Plasma Samples

The blood samples were provided by the Astrid Fagraeus Laboratory, Karolinska Institutet, Stockholm, Sweden, under the ethical permit No. 9544–2019. Samples were collected in 4 mL EDTA tubes (BD Diagnostics), kept at room temperature and processed within a few hours after collection. EDTA tubes were first centrifuged at 200*g* at room temperature for 10 min. The plasma was transferred to a new Eppendorf microtube and centrifuged at 1000*g* at room temperature for 10 min. Plasma was aliquoted into fresh Eppendorf microtubes and frozen at −80 °C until analysis.

### High Abundant Protein Depletion

#### Agilent Plasma 14 Multiple Removal System

Agilent Plasma 14 Multiple Removal System 4.6 × 100 Hu-14 was set up on an Agilent HPLC system (Agilent technologies), 40 μL of plasma were applied to each injection and run according to the manufacturer’s instructions. The depleted plasma flow-through was concentrated on 5 kDa molecular weight cut off filter followed by buffer exchange to 50 mM HEPES pH 7.6 for the TMT-analysis.

#### High Select Top14 Abundant Protein Depletion Mini Spin Columns and Midi Columns

Depletions were performed according to the manufacturer’s recommendations.

Briefly, 10 μL of plasma were applied to each Mini column and 40 μL of plasma were applied to each Midi column, respectively, and incubated at room temperature with gentle end-over-end mixing, for 20 min. Depleted flowthroughs were recovered by centrifugation. The depleted plasma flow-through was concentrated on 5 kDa molecular weight cut off filter followed by buffer exchange to 50 mM HEPES pH7.6 for the TMT-analysis.

#### Heat Treatment

Three aliquots of crude plasma samples from one healthy donor were heated at 56 °C for 30 min prior to depletion. In parallel, three depleted aliquots from the same individual were also heated postdepletion for 30 min at 56 °C.

#### QC of Depleted Plasma Samples

After protein concentration measurement, quality check was applied on all the depleted samples by using ThermoFisher Scientific NuPAGE protein gel system. Ten micrograms of protein of each sample were loaded to the gel (Supporting Figure S4)

### MS Sample Preparation

#### Digestion and Labeling

Depleted plasma was denatured at 60 °C for 1 h followed by reduction with DTT at 95 °C for 30 min and alkylation with chloroacetamide at room temperature for 20 min at end concentrations of 4 mM. Trypsin was added at a 1:50 (w/w) ratio and digestion was performed at 37 °C overnight. When applicable TMTpro-/TMT-10-plex labeling was performed according to manufacturer’s instructions. Labeling efficiency was evaluated by LC-MS/MS on individual samples using 30 min gradients to ensure >95% labeling of peptides before pooling. Following digestion (and labeling if applicable), 1 mL Strata X-C 33u columns (Phenomenex) were used for sample cleanup. The peptides were subsequently dried in a speedvac.

#### HiRIEF Separation

HiRIEF was performed as previously described.^10^ Briefly, the samples were rehydrated in 8 M urea with bromophenol blue and 1% IPG buffer, and subsequently loaded to the immobilized pH gradient (IPG) strip and run according to previously published isoelectric focusing (IEF) protocols.^10^ After IEF, the IPG strip was eluted into 72 fractions using in-house robot. The obtained fractions were dried using SpeedVac and frozen at −20 °C until MS analysis.

#### LC-ESI-MS/MS Q-Exactive HF

Online LC-MS was performed as previously described^10^ using a Thermo UltiMate 3000 RSLCnano System coupled to a Q-Exactive-HF Hybrid Quadrupole-Orbitrap mass spectrometer (Thermo Scientific). Each of the 72 plate wells was dissolved in 20 μL solvent A and 10 μL were injected. Samples were trapped on a C18 guard-desalting column (Acclaim PepMap 100, 75 μm × 2 cm, nanoViper, C18, 5 μm, 100 Å), and separated on a 50 cm long C18 column (Easy spray PepMap RSLC, C18, 2 μm, 100 Å, 75 μm × 50 cm). The nano capillary solvent A was 95% water, 5% DMSO, 0.1% formic acid; and solvent B was 5% water, 5% DMSO, 95% acetonitrile, 0.1% formic acid. At a constant flow of 0.25 μL min^–1^, the curved gradient went from 6 to 10% B up to 40% B in each fraction in a dynamic range of gradient length (see Supporting Table S1), followed by a steep increase to 100% B in 5 min.

FTMS master scans with 60 000 resolution (and mass range 300–1500 *m*/*z*) were followed by data-dependent MS/MS (30 000 resolution) on the top 5 ions using higher energy collision dissociation (HCD) at 30% normalized collision energy. Precursors were isolated with a 2 *m*/*z* window. Automatic gain control (AGC) targets were 1 × 10^6^ for MS1 and 1 × 10^5^ for MS2. Maximum injection times were 100 ms for MS1 and 400 ms for MS2. The entire duty cycle lasted ∼2.5 s. Dynamic exclusion was used with 30 s duration. Precursors with unassigned charge state or charge state 1 were excluded. An underfill ratio of 1% was used.

#### Data Searches

Orbitrap raw MS/MS files were converted to mzML format using msConvert from the ProteoWizard tool suite.^17^ Spectra were searched using the ddamsproteomics pipeline (v1.5) (DOI 10.5281/zenodo.3714589), which is a Nextflow^18^ pipeline runningMSGF+ (2020.03.12),^19^ Percolator (v3.4).^20^ All human searches were done against the human protein coding subset of Ensembl version 92 (107 844 entries), and macaque samples were searched against UniProt cynomolgus macaques protein sets (2020–10–01, 46345 entries). MSGF+ settings included precursor mass tolerance of 10 ppm, fully tryptic peptides, maximum peptide length of 50 amino acids and a maximum charge of 6. Fixed modifications were TMTpro 16 plex (depletion data) or TMT 10-plex (heat-inactivation data) on lysines and peptide N-termini, and carbamidomethylation on cysteine residues, a variable modification was used for oxidation on methionine residues. Quantification of TMTpro 16 plex reporter ions was done using OpenMS project’s IsobaricAnalyzer (v2.5).^21^ PSMs found at 1% FDR (false discovery rate) were used to infer gene identities.

Protein quantification by TMTpro 16 plex reporter ions was calculated using medians of log2-transformed PSM channel intensities from which were subtracted the average value of all channels per PSM. Protein and gene quantification values were then normalized by subtracting their channel medians. Protein false discovery rates were calculated using the picked-FDR method using gene symbols as protein groups and limited to 1% FDR^22^

### Statistical Analyses

Statistical analyses were performed using R (version 4.0.3, R Core Team, 2017, https://www.R-project.org/) and R Studio (version 1.3.1093, RStudio Team 2015, http://www.rstudio.com/). For analysis of differential protein levels between samples we applied Limma within the DEqMS package^23^ (version 1.6.0), https://bioconductor.org/packages/release/bioc/html/DEqMS.html) in R. Correction for multiple testing was performed using the Benjamini–Hochberg method.

Correlations and associated *p*-values (Spearman and Pearson) were calculated using stat_cor in R. Hierarchical clustering analysis, was performed using Pearson correlation, and the results visualized in heatmaps using the R-package pheatmap (version 1.0.12, https://CRAN.R-project.org/package=pheatmap). Principal components analysis was performed using either SIMCA software (version 16.0.2, Umetrics, https://umetrics.com/kb/simca-16), or in R using prcomp, and the factoextra package (1.0.7, https://CRAN.R-project.org/package=factoextra) for extracting and visualizing the results. The PCA was performed on log2 TMT-ratio intensities. Unit variance scaling was applied. Descriptive statistics (violin plots and bar charts) were performed using the software GraphPad Prism (version 9.0.1, https://www.graphpad.com).

## Results

### Evaluation of Spin Columns vs HPLC Columns

The overall aim of the current project was to explore if single-use spin columns for high abundant plasma protein depletion would provide similar depletion efficacy and robustness as traditional HPLC columns, but at an increased throughput. The MARS-14 HPLC column (Agilent; from here on denoted “MARS”), targeting 14 proteins (Table 1), was chosen as gold standard and compared with two spin columns with different loading capacity, High Select Top14 Abundant Protein Depletion Midi Spin Column (from here on denoted “Midi”) and High Select Top14 Abundant Protein Depletion Mini Spin Column (from here on denoted “Mini”), both Thermo Scientific (Table 1).

**Table 1:** Summary of Number of Identified Proteins, Peptides, and PSM

	plasma depletion procedure							LCM5/MS						
experiment	commercial products	company	loading capacity	number of use(s)	capturing agents	ideally captured proteins		operation	labeling	HiRIEF pH	depleted plasma (mg)	# PSM	# peptides	# proteins
Set 1^a^	High Select	ThermoFisher Scientific	Up to 10 μL (load 10 μL)	Single	Antibodies	Albumin	Alpha-1-acidglycoprotein	Centrifuge^c^	TMTpro 16-plex	3–10	0.72^e^	101 725	16347	1913
	Top14					IgA	Alpha-1-antitypsin							
	Abundant					IgD	Alpha-2-macroglobulin							
	Protein					IgE	Apolipoprotein a A1							
	Depletion					IgG	Fibrinogen							
	Mini Spin					IgG (light chain)	Haptogloblin							
	Columns					IgM	Transferrin							
Set 2^a^	High Select	ThermoFisher Scientific	Up to 10 μL (load 10 μL)	Single	Antibodies	Albumin	Alpha-1-acidglycoprotein	Centrifuge^c^	TMTpro 16-plex	3–10	1.04^e^	119 395	16449	1897
	Top14					IgA	Alpha-1-antitypsin							
	Abundant					IgD	Alpha-2-macroglobulin							
	Protein					IgE	Apolipoprotein a A1							
	Depletion					IgG	Fibrinogen							
	Mini Spin					IgG (light chain)	Haptogloblin							
	Columns					IgM	Transferrin							
Set 3^a^	Multiply	Agilent	30–70 μL (load 10 μL)	Multiple (>200 Injections)	Antibodies	Albumin	Alpha-1-acid glycoprotein	HPLC^d^	TMTpro 16-plex	3–10	0.96^e^	109 157	16360	1878
	Affinity					IgA	Antitypsin							
	Removal					IgG	Alpha-2-macroglobulin							
	Column					IgM	Apolipoprotein a A1							
	Human 14					Fibrinogen	Apolipoprotein a A2							
						Haptogloblin	Complement C3							
						Transferrin	Transthyretin							
Set 4^b^	High Select	ThermoFisher Scientific	Up to 10 μL (load 10 μL)	Single	Antibodies	Albumin	Alpha-1-acid glycoprotein	Centrifuge^c^	TMTpro 10-plex	3–10	0.48^e^	64900	12854	2039
	Top14					IgA	Alpha-1-antitypsin							
	Abundant					IgD	Alpha-2-macroglobulin							
	Protein					IgE	Apolipoprotein a A1							
	Depletion					IgG	Fibrinogen							
	Mini Spin					IgG (light chain)	Haptogloblin							
	Columns					IgM	Transferrin							

a The same 14 human plasma samples plus duplicates of the internal standard.

b The same individual female plasma sample with PSA: w/o heating ×4, depletion ×3, heating after depletion ×3.

c Avg. 40 min per 14 samples.

d 40 min per sample, plus buffer wash (40 min) between two samples.

e Load half to LC-MS/MS.

First, we wanted to explore if the spin columns could provide the same number of identified proteins as the standard HPLC column. We decided to evaluate two spin columns with identical depletion resins, but with different loading capacity: one with a maximum load of 100 μL plasma (Midi), and one with maximum load of 10 μL human plasma (Mini). The potential advantage of the Mini column would be the microcentrifuge format and the reduced sample volume needed. To make a head-to-head comparison we loaded the same amount of plasma (40 μL) on both the MARS-14 column and the Midi spin column (Since we have previously shown that increased protein load is correlated to high number of identified proteins^10^) and 10 μL on the Mini column.

To account for individual variability, an identical set of 14 plasma samples + two pooled internal standards from 14 different individuals were depleted using each column and then subjected to downstream in-depth plasma HiRIEF LC-MS/MS using 16-plex TMT labeling as previously described^10^ (set 1–set 3, Table 1). On average the protein yield from the Mini, Midi and MARS-14 was 59, 208, and 137 μg, respectively (Supporting Table S2). Depletion time for each sample using the MARS-14 column on a HPLC system was 40 min (effective depletion time, not including the setup, wash). In comparison, since several samples can be prepared in parallel using the spin columns, each TMT-16 plex set with 16 samples could be depleted during the same time frame using the spin columns, highlighting the benefit of using single-use spin columns for increased throughput.

In total 1884 proteins (protein centric, 1% peptide and protein FDR) were detected using the standard MARS-14 depletion approach loading close to 1 mg total peptide on the HiRIEF strip, which is in line with the number of proteins detected per set in previous analyses using same method.^10,24^ From the Midi spin column 1905 proteins (1% peptide and protein FDR, 1.06 mg peptide on strip) were identified and from the Mini spin columns 1931 proteins (1% peptide and protein FDR, 0.72 mg peptide on strip) (Table 1). This initial analysis showed that a similar number of identifications could be obtained using the spin columns as with the traditional HPLC column and that reducing the sample load from 40 μL crude plasma to 10 μL plasma did not have a negative effect on the number of identified proteins.

**Figure 2:**
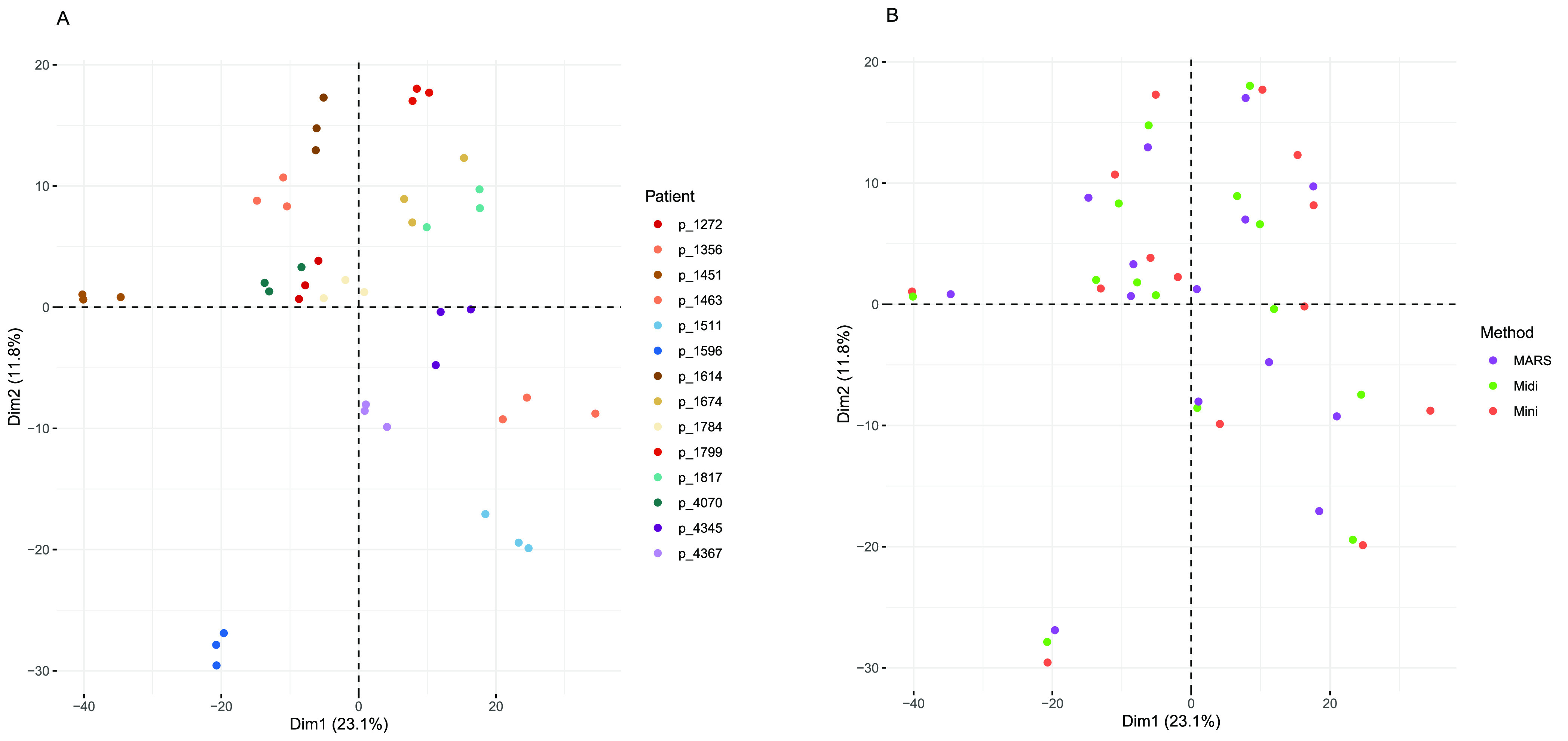
Principal components analysis (PCA) performed on data from the plasma depletion column study in which triplicate plasma samples from 14 patients with lung-cancer were depleted by High Select Top14 Abundant Protein Depletion Mini Spin Column (“Mini”), High Select Top14 Abundant Protein Depletion Midi Spin Column (“Midi”; both Thermo Scientific), or by MARS-14 HPLC column (Agilent), targeting 14 proteins (“MARS”). The PCA scores plot shown for the first two components explain approximately 33% of the variance in the data. (A) Coloring of samples according to individual from which the blood was drawn. (B) Coloring of samples according to plasma depletion method (Mini, Midi or MARS). All samples (*n* = 42) and all variables detected in at least 50% of the samples were included in the analysis (*n* = 1811, 1% peptide and protein FDR).

As the MARS column and the Mini and Midi columns target a slightly different set of proteins (11 of 14 overlapping), we wanted to explore if the different depletion methods introduced depletion specific batch effect on the samples. To get an overview of sample similarity/dissimilarity based on protein expression, principal components analysis was performed (Figure 2).

Judging from the PCA scores plot, it was evident that the samples clustered by individual rather than by depletion method, showing that the type of depletion system did not greatly affect the data overall. Instead, the individual feature of each sample was well preserved throughout the three set ups, showing the robustness of the depletion approach in general.

To further explore the agreement between the three different depletion methods, the correlations between the plasma depletion methods were pairwise compared (Mini versus Midi, Mini versus MARS and Midi versus MARS, respectively) protein by protein for each of the samples. Spearman’s correlation coefficients (*R*^2^) and statistical significance were calculated from the points. In brief, median *R*^2^ was for Mini vs Midi: 0.67 (range: 0.63–0.84); Mini vs MARS 0.68 (range: 0.56–0.83); and for Midi vs MARS: 0.60 (range: 0.51–0.85), Supporting Figure S1, showing that the overall agreement was similar between the individual methods regardless of which column was used.

**Figure 3:**
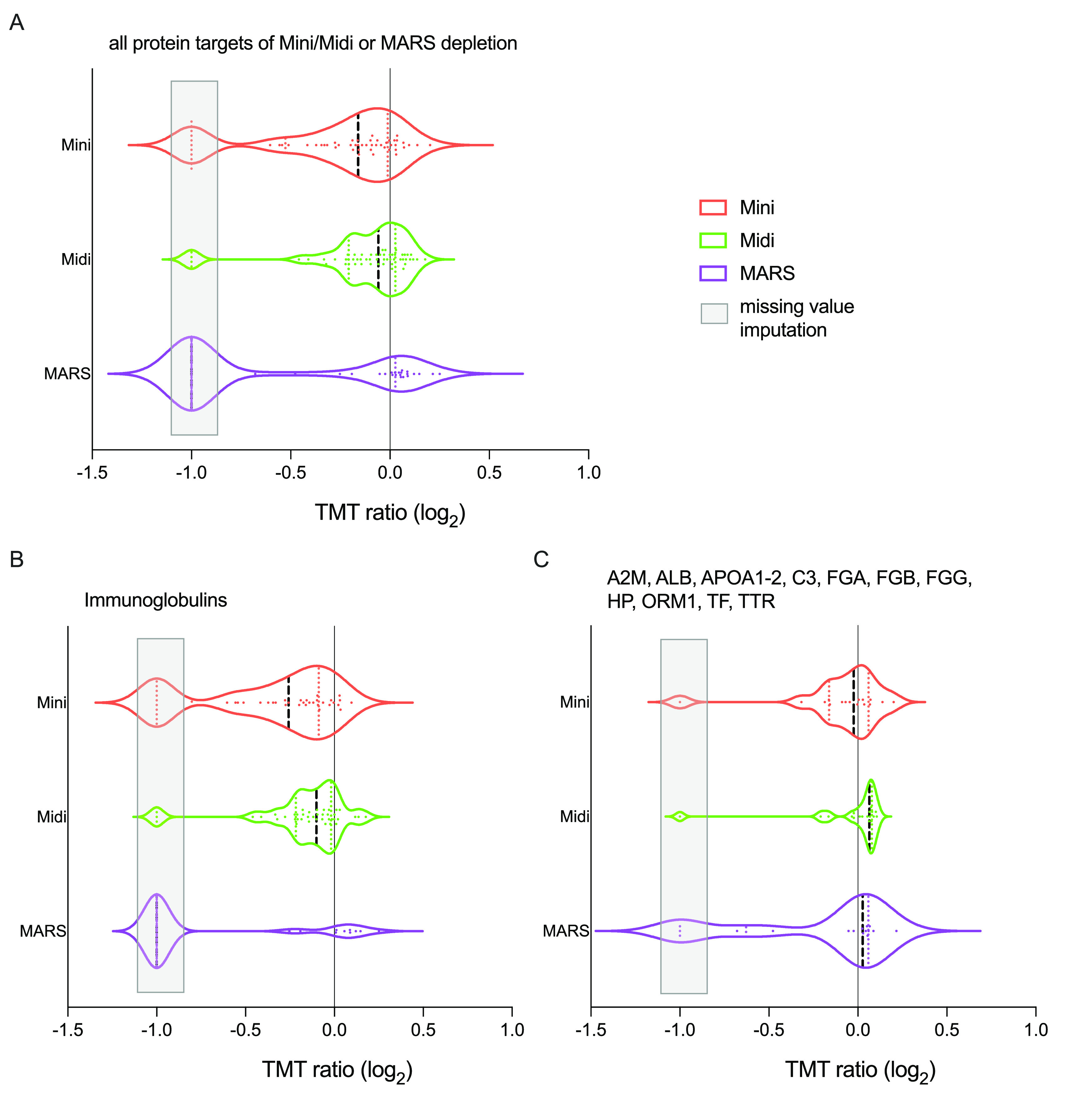
Violin plots of the protein expression of the “depleted” proteins, for comparing the performance of the depletion of high-abundant plasma proteins with either of the three methods. Protein expression as TMT relative rations (log_2_) are shown for (A) all proteins targeted by any of the depletion methods, and the depletion targets subgrouped into (B) immunoglobulins and (C) the remaining depletion targets. Missing values, presumably indicating proteins removed by the depletion were imputed “–1” (light gray box).

Given that the depletion columns target a slightly different set of proteins (Table 1), we wanted to further explore if the specific proteins targeted by the depletion were altered between the different set-ups. To this end, we visualized the proteins targeted by either of the depletion methods by violin plots as shown in Figure 3. Subdividing the targeted proteins into immunoglobulins (Figure 3B) and other plasma protein targets (Figure 3C), we could see that the methods differed in the depletion of immunoglobulins (Figure 3B,C). By visualizing the individual protein expression for each method in boxplots we could see that among the proteins targeted exclusively by the MARS column (gene names C3, TTR and APOA2), C3 was below detection level for 2 out of 4 protein isoforms in the MARS-depleted samples, showing high degree of variability in the effectiveness of depletion between the individual isoforms (Supporting Figure S2). APOA2 levels were also reduced, but to a varying degree between the samples. Among the proteins listed as targets of depletion uniquely for the Mini/Midi column (IgE, IgD, and IgG light chains), it was evident that these were also depleted and even so to a larger extent, by the MARS column. However, it is worth noticing that the MARS columns target the IgG protein, including both heavy and light chains. The levels of IgG kappa and lambda light chains were in almost all cases below detection limit in the MARS depleted samples. The proteins targeted by all three methods were removed equally well across all three methods.

Overall, these comparisons showed that the depletion columns performed equally well in terms of number of identified proteins in the depleted samples (Table 1), and that neither of the depletion columns had a major impact on the global protein expression pattern of the samples (Figure 2). Hence, we chose to move forward with the Mini column, since it required less material and was easy to use in a normal table-top microcentrifuge.

### Effect of Heat Treatment for Virus Inactivation

The COVID-19 pandemic has highlighted the need of performing viral inactivation of potentially infected plasma samples. Heat inactivation is an appealing simple and effective measure to inactivate the SARS-CoV-2 virus^25^ and is commonly used to inactivate other enveloped viruses. However, heat inactivation of crude plasma samples could potentially affect the depletion efficacy negatively, by altering the epitopes though protein denaturation during the heating procedure. An alternative approach would be to perform the depletion of infected samples in an BSL3 environment and then perform the heat inactivation on depleted plasma. In this setting, the microcentrifuge spin columns have several advantages as the centrifuge can easily be placed in a BSL3 environment and single-use columns are easy to disregard after exposure to potentially infected material. Hence, we set out to test the effect of heat inactivation, before (from here on denoted “preHeat”) and after high abundant protein depletion (from here on denoted “postHeat”) using the spin microcolumn and compare with the standard protocol (from here on denoted “noHeat”). Heat inactivation at 56 °C for 30 min was chosen, as this has previously shown to inactivate the SARS-CoV-2 virus, with minimal impact on serological analysis.^25^ The experiment was performed in triplicates using plasma from a healthy donor. In total 2062 proteins (1% protein and peptide FDR, Table 1) was detected in the experiment.

**Figure 4:**
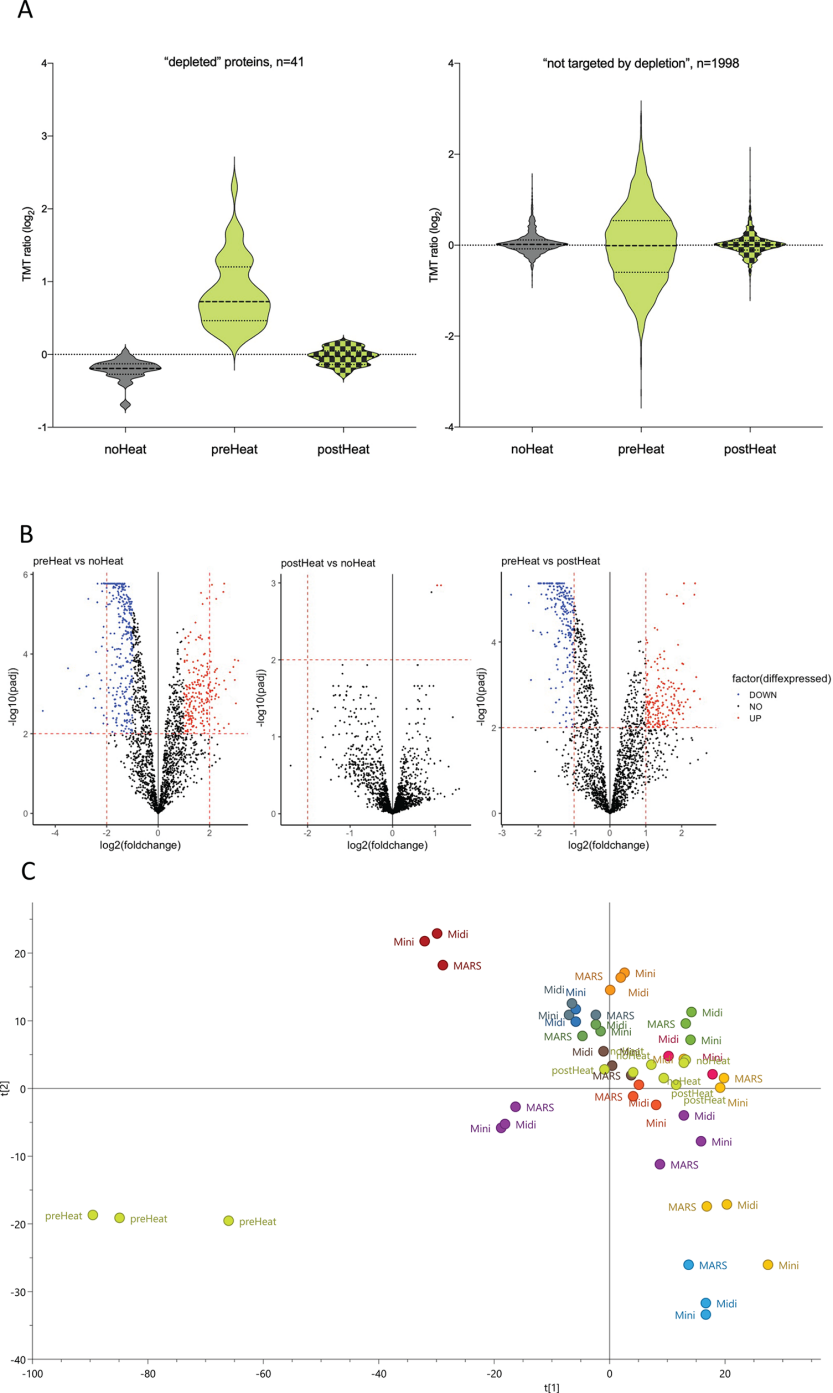
Impact of heat-inactivation treatment on depletion efficacy. (A) Violin plots displaying the expression intensity distribution of the proteins within the heat-inactivation study. The violin plots are filled in gray (left) for no heat-inactivation (“noHeat”), light green (middle) for heat-inactivation prior to depletion (“preHeat”), and light green with fill pattern (right) for depletion after heat-inactivation of samples (“postHeat”), respectively. The left figure shows the intensity distribution of proteins targeted by the depletion method and with measurable levels (*n* = 41), as listed in Table 1. For protein identities (gene name) of depleted proteins and their individual expression, see Supporting Figure S3. The right figure shows the intensity distribution of all other proteins detected (*n* = 1998). (B) Differential expression analysis comparing plasma proteome profiles from plasma depleted without, before, and after heat-inactivation. Replicate plasma samples from the same individual was used. (C) Principal components analysis (PCA), performed on data from both the depletion column study and the heat-inactivation study.

To investigate whether the depletion efficacy was affected by the heat treatment, we plotted the expression data of the proteins supposedly removed in the depletion step (listed in Table 1, Mini Spin column). We also plotted the remaining bulk of proteins to see whether the observed differences were limited to the “depleted proteins” or an overall effect on all proteins. It was apparent that the difference between the heat-treatment groups was related to the set of “depleted” proteins, we could conclude that the efficacy of the depletion was negatively affected by the heat-inactivation step (Figure 4A left). The preHeat samples also showed a larger variability overall (Figure 4A right), possibly due to precipitation caused by the heating hampering the effectiveness of the depletion, allowing high abundant proteins through and thereby negatively affecting the detection of other lower abundant proteins. In addition, a differential expression analysis showed no major differences when comparing postdepletion heat inactivation and no-heat inactivation plasma profiles, but major differences when comparing predepletion heat inactivation and no-heat-inactivation plasma profiles (Figure 4B). To visualize the magnitude of the effect of heat inactivation in relation to the known individual differences in protein expression, we performed PCA analysis including all samples analyzed in the current study (Figure 4C). Judging from the PCA, the samples again cluster together regardless of depletion method, and regardless of whether noHeat and postHeat. However, the preHeat samples appeared different from the rest. Taken together, it is clear that heating the crude plasma samples has a major effect on the depletion efficacy, and hence also the downstream proteomics results, and should be avoided. Heat inactivation after the depletion, on the other hand, has minimal effect on the proteome and could easily be performed in a BSL3 environment.

### Cross Species Compatibility

**Figure 5:**
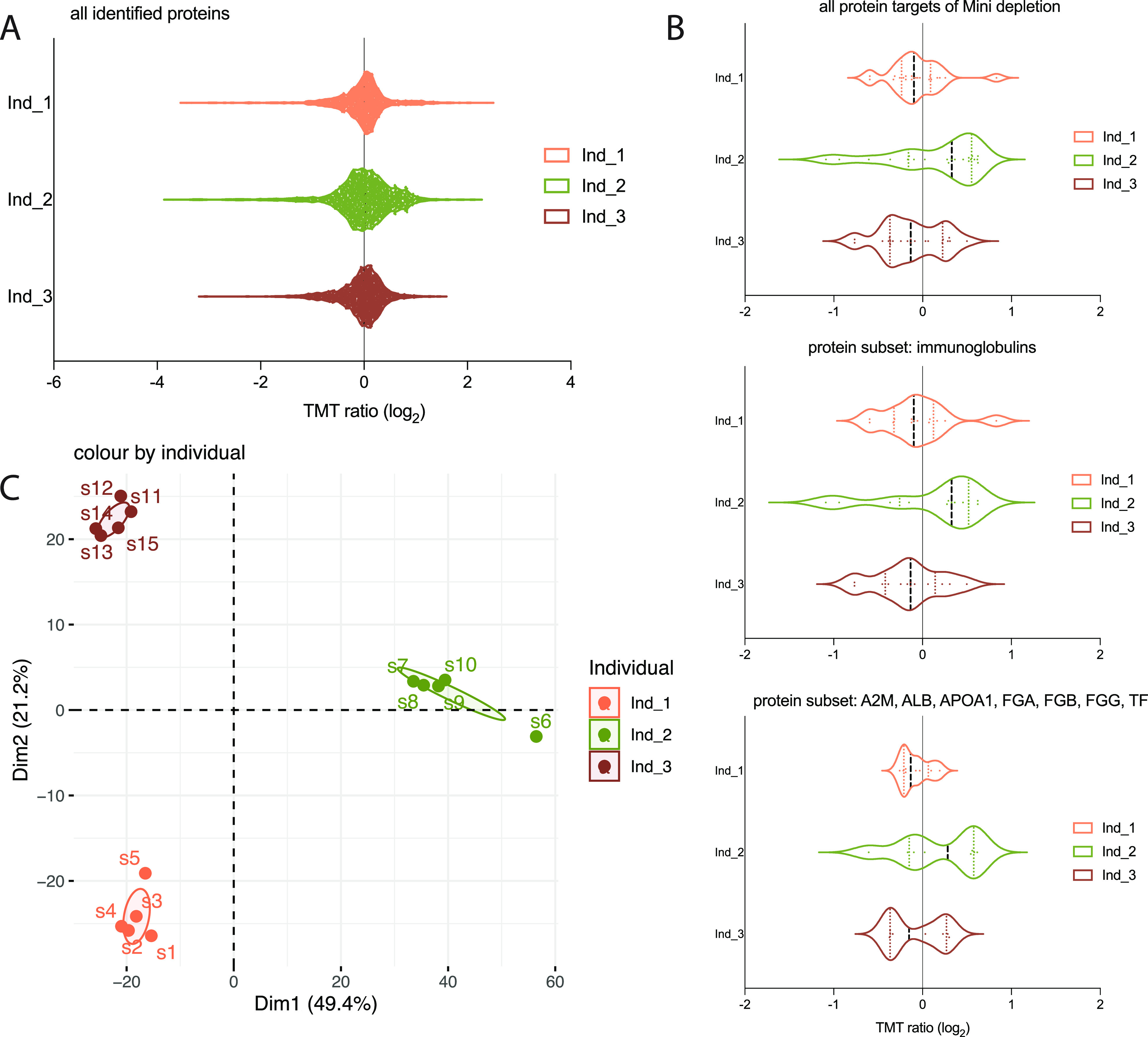
Cross species compatibility and reproducibility. The Mini depletion spin columns were applied on 5-plicate plasma samples from 3 *Macaca fascicularis*. (A) Violin plots showing protein expression distribution of total proteome, (B) all protein targets of depletion and all protein targets of depletion subdivided into immunoglobulins and other plasma proteins, respectively. (C) Principal components analysis (PCA) applied to the proteomics expression data.

In vaccine and drug development, animal models like cynomolgus macaques (*Macaca fascicularis)* are commonly used in the early phases to test for efficacy and toxicity. Macaque and human have a high degree of sequence homology and hence, we wanted to explore if the depletion spin columns were compatible with plasma from *Macaca fascicularis.* Five replicates of plasma samples from 3 healthy monkeys, including a pooled internal standard, were depleted by High Select Top14 Abundant Protein Depletion Mini Spin Columns and included in a TMTpro 16 plex-HiRIEF set. Prior to digestion we separated the depleted samples on SDS-page gels to get an overview of the depletion efficacy, here the depleted *Macaca* samples showed similar patterns as seen on gels with human depleted plasma samples, which was encouraging (Supporting Figure S4, GEL IMAGES). Indeed, in the downstream analysis, in total 2380 proteins (1% protein and peptide FDR) were identified. Looking closer at the targeted proteins, the effectiveness of the depletion was also seen in the MS data (Figure 5). Overall, this shows that the High Select Top14 Abundant Protein Depletion Mini Spin Columns are compatible with plasma from *Macaca fascicularis*.

### Reproducibility

Reproducibility and robustness of the method is of outmost importance in plasma proteomics. We have previously shown that when using the plasma HiRIEF LC-MS/MS protocol we could obtain technical coefficient of variation (CV) of 4.7% on peptide level, *not* including the depletion, buffer exchange, or digestion steps. On the basis of the replicate *Macaca fascicularis* samples (5 per individual animal) we also calculated the CV within each individual animal to 11, 14, and 11 CV%, respectively. To further explore the reproducibility, the correlation between the individual replicates were calculated for each of the animals. Spearman’s correlation coefficients (*R*^2^) and statistical significance was calculated from the points. In brief, median *R*^2^ was 0.82 (range: 0.70–0.96) Supporting Figure S5, again showing high reproducibility of the workflow. This shows that the depletion, buffer exchange, and digestion steps together contribute to a large proportion of the variability; however, the overall variability of the entire workflow is still low.

## Conclusion

The overall aim of the current study was to evaluate if single-use spin columns for high abundant protein depletion could provide a simple and time-efficient alternative to standard HPLC columns, without compromising the analytical depth and throughput. Second, we also wanted to evaluate if the spin columns were compatible with viral inactivation through heating and compatible with depletion of plasma from macaque.

Here we show that the depleted plasma from the spin columns can be subjected to viral deinactivation by heating postdepletion without negatively effecting the proteome coverage or reproducibility. As the spin columns do not require any advanced equipment or handling, the depletion can therefore easily be performed within the BSL3 setting. This is of importance also for studies of other pathogens that can be deactivated through heating. We also show that the proteome coverage using the micro spin columns is on par with traditional multiple-use HPLC columns and provides a flexible and easy-to use alternative. Last, we show that the spin columns are also effectively and reproducibly able to deplete high abundant proteins from plasma samples from macaques, thereby enabling in-depth plasma proteome analysis of this important model animal.

In summary we conclude that the spin columns provide a simple, reproducible, and cost-effective way to perform high abundant protein depletion prior to in-depth MS based plasma analysis.

## Supporting Information Available

Figure S1: Correlation between the 3 different plasma depletion methods; Figure S2: Comparing the performance of the depletion of high-abundant plasma proteins with either of the three methods, boxplots per protein; Figure S3: Heat-inactivation cohort, protein expression of “depleted” proteins; Figure S4: SDS-PAGE gel images showing depletion efficacy; Figure S5: Correlation matrix; Table S1: HiRIEF fractions gradient length; Table S2: Protein yield after depletion (PDF)

## Notes

For publication, all RAW MS-based proteomics data were deposited to the ProteomeXChange Consortium (http://proteomecentral.proteomexchange.org/cgi/GetDataset) via the PRIDE partner repository and accessed with the identifier PXD023148.
