# Supplementary material for: Evaluation of Spin Columns for Human Plasma Depletion to Facilitate MS-Based Proteomics Analysis of Plasma
Source: J Proteome Res. 2021 Jul 28;20(9):4610–20. doi: 10.1021/acs.jproteome.1c00378 (PMC8419864; doi:10.1021/acs.jproteome.1c00378)
Supplement: Supplementary file 1 — pr1c00378_si_001.pdf [file pr1c00378_si_001.pdf]

SUPPLEMENTARY MATERIAL

Figure S1. Correlation between the 3 different plasma depletion methods.

Figure S2. Comparing the performance of the depletion of high-abundant plasma proteins with either of the three methods. Boxplots per protein.

Figure S3. Heat-inactivation cohort, protein expression of “depleted” proteins.

Figure S4: SDS-PAGE gel images showing depletion efficacy

Figure S5: Correlation matrix

Table S1: HiRIEF fractions gradient length.

Table S2: Protein yield after depletion

SUPPLEMENTARY FIGURES

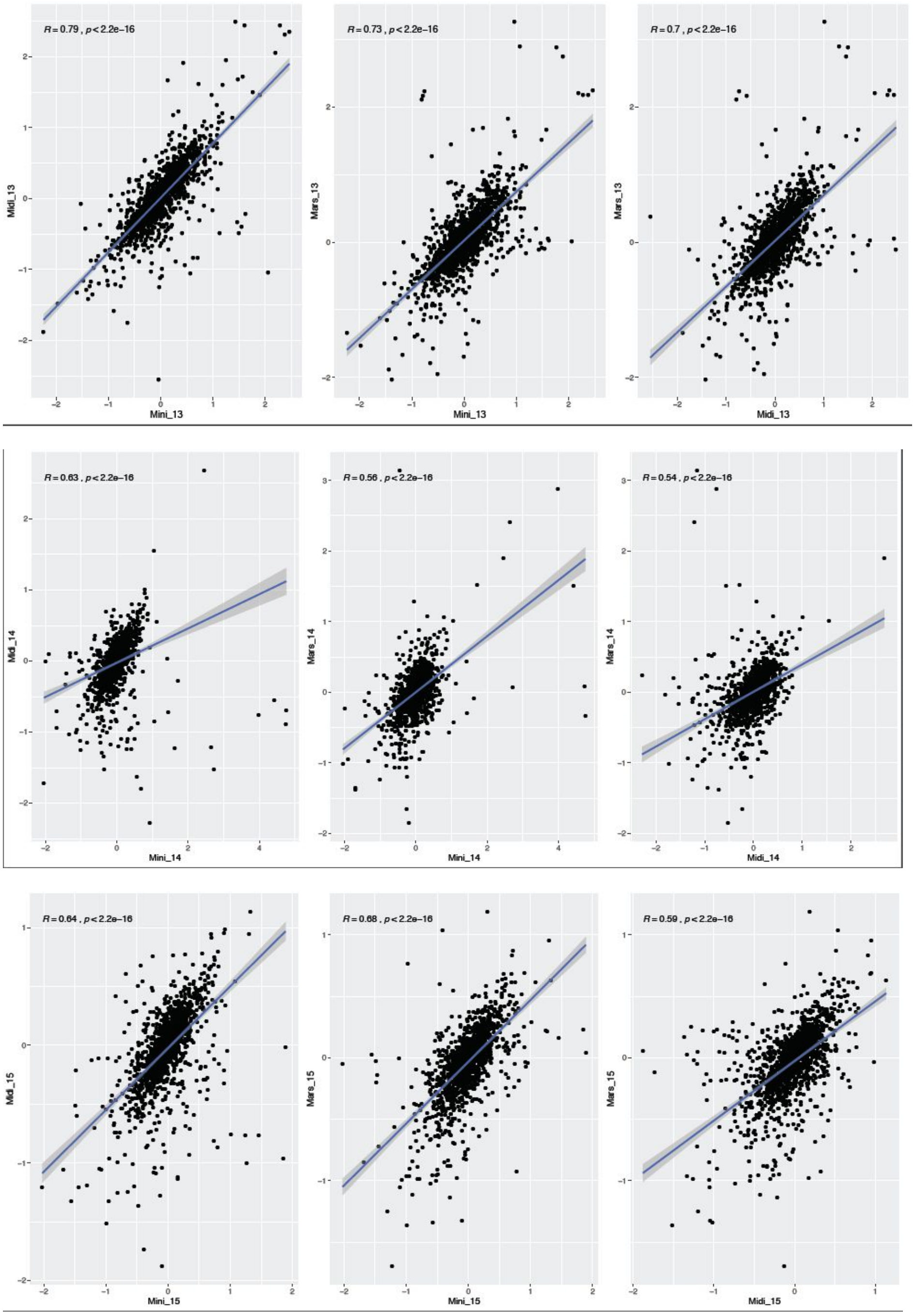

(in total 2x14 plots, separate .pdf file, both Spearman and Pearson correlation)

Supplementary Figure 1. Correlation between the 3 different plasma depletion methods. Correlation plots show pairwise comparisons (Mini versus Midi, Mini versus MARS and Midi versus MARS, respectively) protein by protein for each of the samples (named 13-26). Spearman´s correlation coefficients (R^2) and statistical significance was calculated from the points. In brief, median R^2 was for Mini vs Midi: 0.67 (range: 0.63-0.84); Mini vs MARS 0.68 (range: 0.56-0.83); and for Midi vs MARS: 0.6 (range: 0.51-0.85).

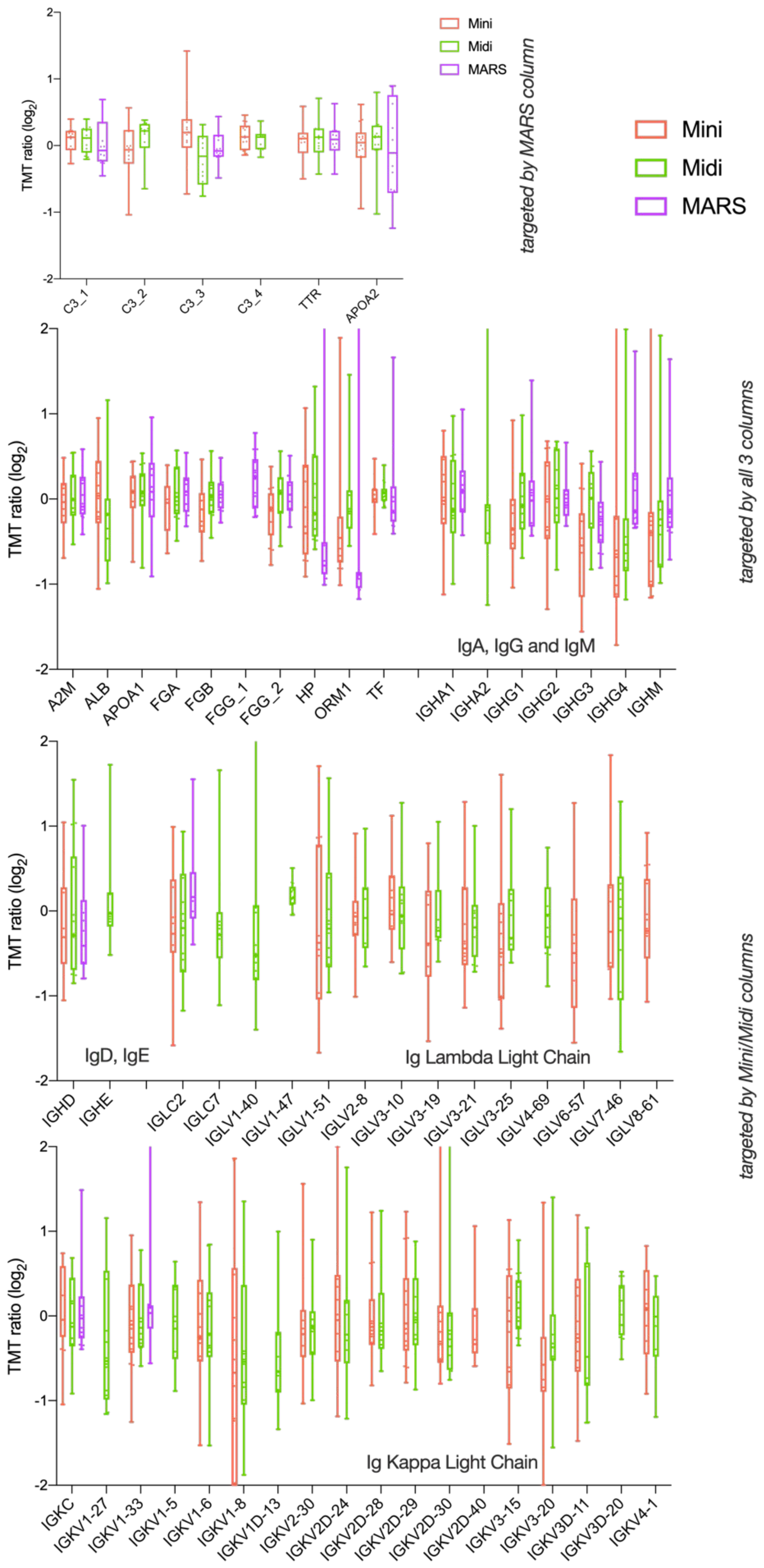

Supplementary Figure 2. Comparing the performance of the depletion of high-abundant plasma proteins with either of the three methods. Boxplots per protein.

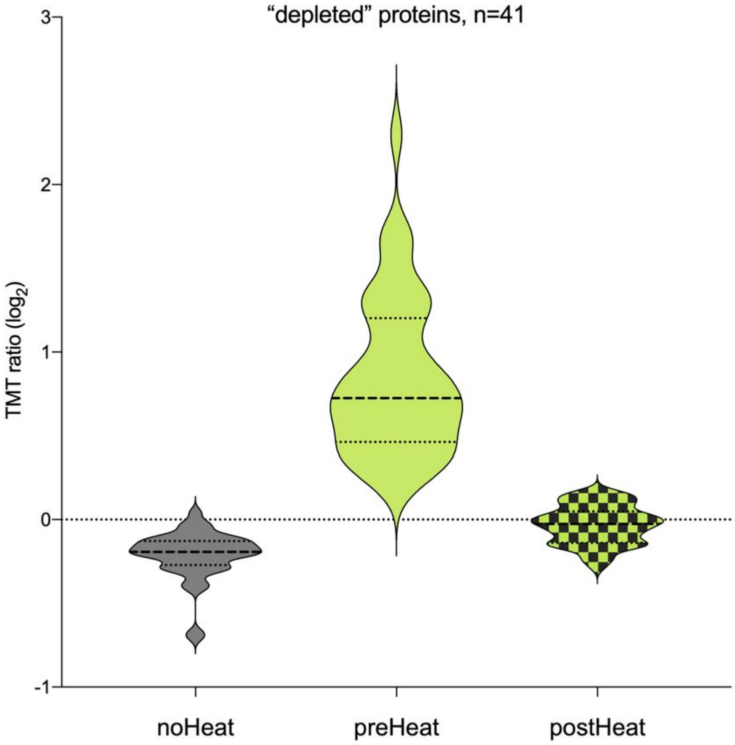

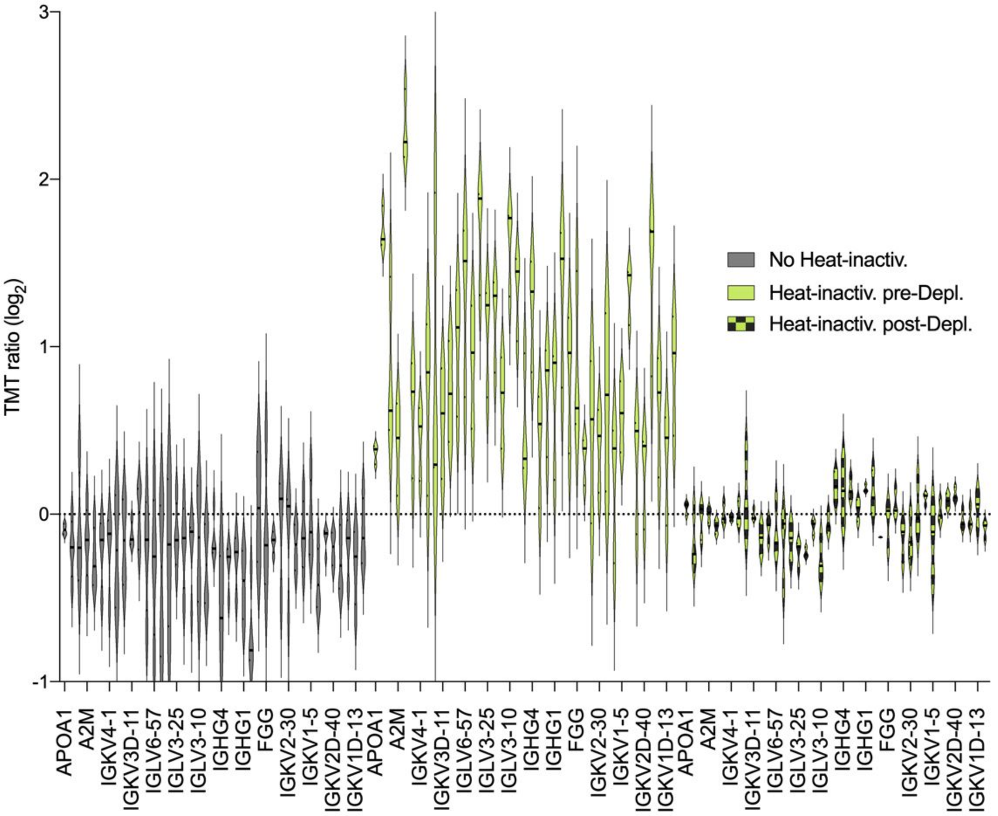

Supplementary Figure 3. Heat-inactivation cohort, protein expression of "depleted" proteins. Same data, two plots.

Human plasma samples

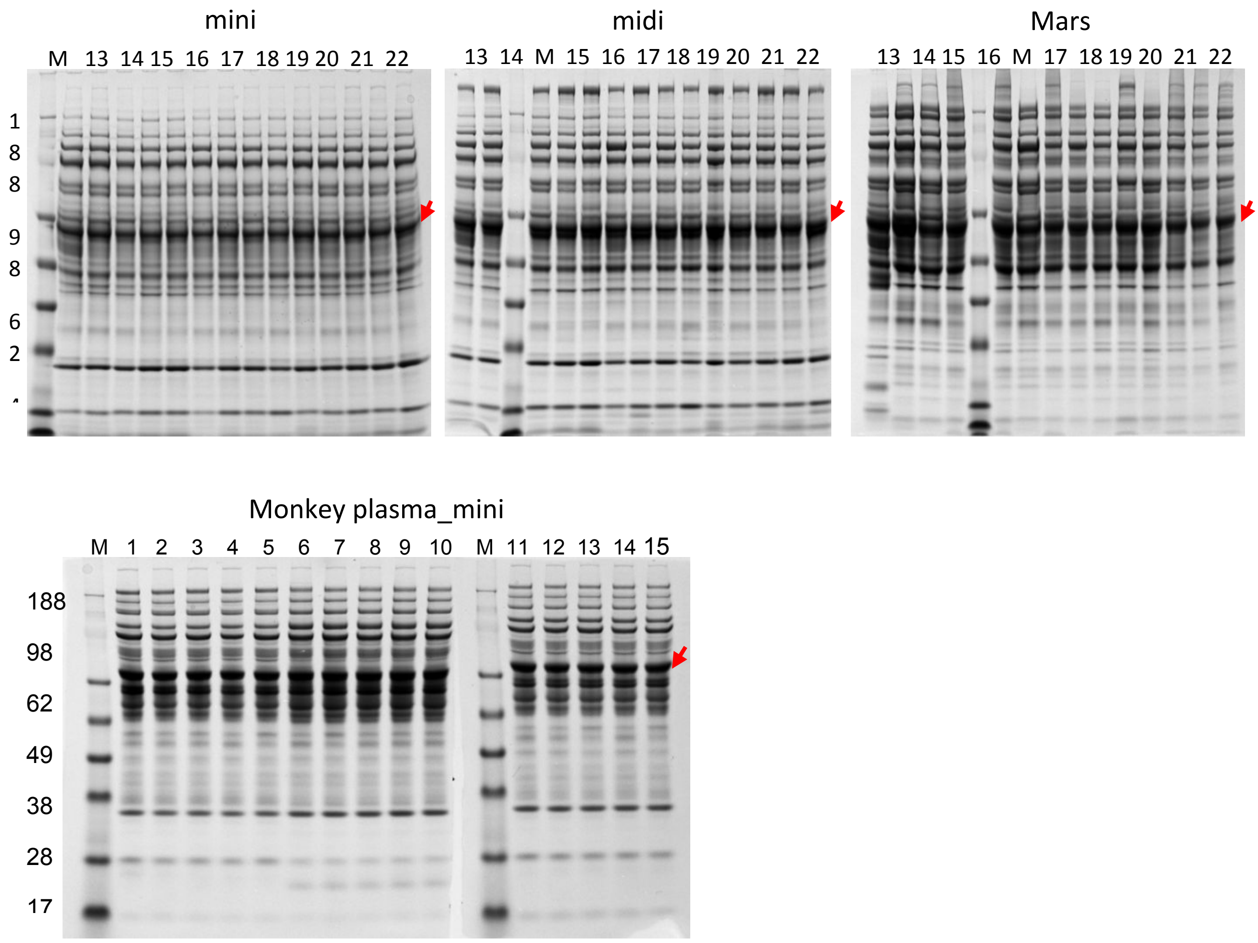

Supplementary Figure 4. NuPAGE™ 4-12% Bis-Tris Protein Gels, 1.5 mm, 15-well, MOPS running buffer Load approximately 10 µg/sample to the PAGE, mini: No.15 only load 5 µg

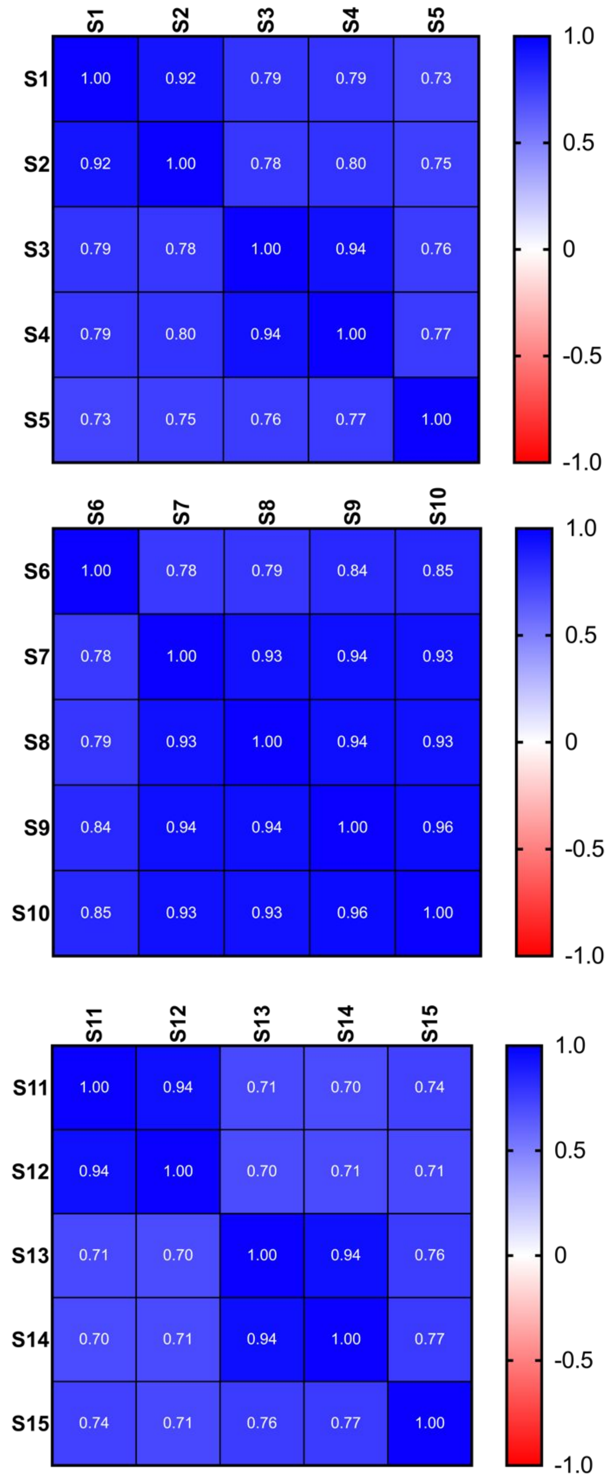

Supplementary Figure 4 Correlation matrices showing spearman correlation between the replicates from the three *Macaca fascicularis*

SUPPLEMENTARY TABLES

Supplementary table 1. HiRIEF fractions gradient length.

Sample	Gradient Length TMT-16	Gradient Length TMT-10
fraction_01	accumulate in trap column	50 min
fraction_02	50 min	50 min
fraction_03	70 min	70 min
fraction_04	70 min	70 min
fraction_05	90 min	90 min
fraction_06	90 min	90 min
fraction_07	110 min	110 min
fraction_08	110 min	110 min
fraction_09	110 min	110 min
fraction_10	110 min	110 min
fraction_11	110 min	110 min
fraction_12	110 min	90 min
fraction_13	110 min	90 min
fraction_14	110 min	90 min
fraction_15	90 min	90 min
fraction_16	70 min	70 min
fraction_17	70 min	50 min
fraction_18	50 min	50 min
fraction_19	50 min	50 min
fraction_20	accumulate in trap column	accumulate in trap column
fraction_21	accumulate in trap column	accumulate in trap column
fraction_22	accumulate in trap column	accumulate in trap column
fraction_23	accumulate in trap column	accumulate in trap column
fraction_24	accumulate in trap column	accumulate in trap column
fraction_25	accumulate in trap column	accumulate in trap column
fraction_26	accumulate in trap column	accumulate in trap column
fraction_27	50 min	50 min
fraction_28	50 min	50 min
fraction_29	70 min	70 min
fraction_30	70 min	50 min
fraction_31	70 min	50 min
fraction_32	70 min	accumulate in trap column
fraction_33	70 min	accumulate in trap column
fraction_34	70 min	accumulate in trap column
fraction_35	70 min	accumulate in trap column
fraction_36	70 min	50 min
fraction_37	70 min	50 min
fraction_38	accumulate in trap column	70 min
fraction_39	accumulate in trap column	70 min
fraction_40	accumulate in trap column	50 min
fraction_41	accumulate in trap column	50 min
fraction_42	accumulate in trap column	50 min
fraction_43	accumulate in trap column	accumulate in trap column
fraction_44	accumulate in trap column	accumulate in trap column
fraction_45	70 min	accumulate in trap column
fraction_46	70 min	accumulate in trap column
fraction_47	70 min	accumulate in trap column
fraction_48	70 min	accumulate in trap column
fraction_49	70 min	accumulate in trap column
fraction_50	50 min	50 min
fraction_51	50 min	70 min
fraction_52	accumulate in trap column	70 min
fraction_53	accumulate in trap column	accumulate in trap column
fraction_54	accumulate in trap column	accumulate in trap column
fraction_55	70 min	accumulate in trap column
fraction_56	50 min	accumulate in trap column
fraction_57	50 min	accumulate in trap column
fraction_58	accumulate in trap column	accumulate in trap column
fraction_59	accumulate in trap column	accumulate in trap column
fraction_60	accumulate in trap column	accumulate in trap column
fraction_61	accumulate in trap column	accumulate in trap column
fraction_62	accumulate in trap column	accumulate in trap column
fraction_63	accumulate in trap column	accumulate in trap column
fraction_64	accumulate in trap column	50 min
fraction_65	accumulate in trap column	50 min
fraction_66	accumulate in trap column	70 min
fraction_67	accumulate in trap column	accumulate in trap column
fraction_68	accumulate in trap column	accumulate in trap column
fraction_69	accumulate in trap column	accumulate in trap column
fraction_70	accumulate in trap column	accumulate in trap column
fraction_71	accumulate in trap column	50 min
fraction_72	50 min	50 min

Supplementary table 2. Yield from depletion

mini-14_set1	Patient.ID	aft de. Prot.	Vol_uL	tot_ug	Average ug
13	1596	0,63	88,2	55,5	**59,3**
14	4070	0,65	88,6	57,6	
15	1784	0,51	88,2	45	
16	1451	0,63	83,2	52,3	
17	1272	0,7	81,7	57,2	
18	1511	0,65	82,6	53,7	
19	1463	0,75	84,7	63,5	
20	1674	0,73	86,3	63	
21	1356	0,79	85,3	67,4	
22	1817	0,83	86	71,3	
23	4345	0,72	81,1	58,4	
24	1614	0,74	78,5	58	
25	4367	0,89	86,8	77,2	
26	1799	0,6	84,3	50,6	

Midi-14_set2	Patient.ID	aft de. Prot. ug/uL	Vol_uL	tot_ug	Avergage ug
13	1596	2,12	110,3	233,8	**208,7**
14	4070	2	117	234	
15	1784	1,48	114,2	169	
16	1451	2,12	99,3	210,4	
17	1272	1,84	98,6	181,4	
18	1511	1,4	97,9	137	
19	1463	2,18	99,4	216,7	
20	1674	1,56	116,6	181,9	
21	1356	1,84	96,6	177,7	
22	1817	2,4	99,8	239,6	
23	4345	2,08	99,2	206,3	
24	1614	2,2	100,5	221	
25	4367	2,48	109	270,2	
26	1799	2,32	104,7	242,9	

Mars14_set 3	Patient.ID	aft de. Prot. ug/uL	Vol_uL	tot_ug	Average ug
13	1596	1,42	87	123,5	**137,1**
14	4070	0,93	78,3	72,8	
15	1784	0,97	93,7	90,9	
16	1451	1,52	833,4	111,6	
17	1272	1,35	85,6	115,6	
18	1511	0,88	85,6	75,4	
19	1463	2,12	76,3	161,7	
20	1674	1,56	89,6	139,8	
21	1356	1,6	82,7	132,4	
22	1817	1,56	69,6	108,6	
23	4345	1,51	26	128,9	
24	1614	2,27	73,6	167,1	
25	4367	3,5	88,1	308,5	
26	1799	2,32	78,7	182,6	
